# Retain in the membrane: Tinkering with the BRX-PAX-PIP5K auxin efflux machinery affects vascular tissue differentiation

**DOI:** 10.1093/plcell/koae058

**Published:** 2024-02-20

**Authors:** Sonhita Chakraborty

**Affiliations:** Assistant Features Editor, The Plant Cell, American Society of Plant Biologists; Department of Forest Genetics and Plant Physiology, Swedish University of Agricultural Sciences, Umeå Plant Science Centre, Umeå, Sweden

The dawn of vasculature in plants more than 400 million years ago was a game changer. This newfound ability of plants to transport water and nutrients over long distances meant that they could grow larger and taller. As young Arabidopsis seedlings develop, 2 protophloem poles harboring protophloem sieve elements (PPSE) and protophloem companion cells are among the first to differentiate (Fig. A) (Roszak et al. 2021). The timely accumulation of the phytohormone auxin in these precursor cells is essential for their differentiation into the phloem of the developing vasculature. Necessary auxin gradients that drive physiological functions are generated by plasma membrane (PM)-integral auxin efflux carrier PIN-FORMED (PIN) as well as the kinases at the PM that affect their polar localization, auxin-efflux capacity, and turnover. Of the PM phospholipids, phosphoinositide phosphatidylinositol-4,5-bisphosphate [PI(4,5)P2] produced by PHOSPHATIDYLINOSITOL-4-PHOSPHATE-5-KINASE (PIP5 K) is important for the recruitment of PM proteins and PIN endocytosis (Barbosa et al. 2016).

**Figure.**
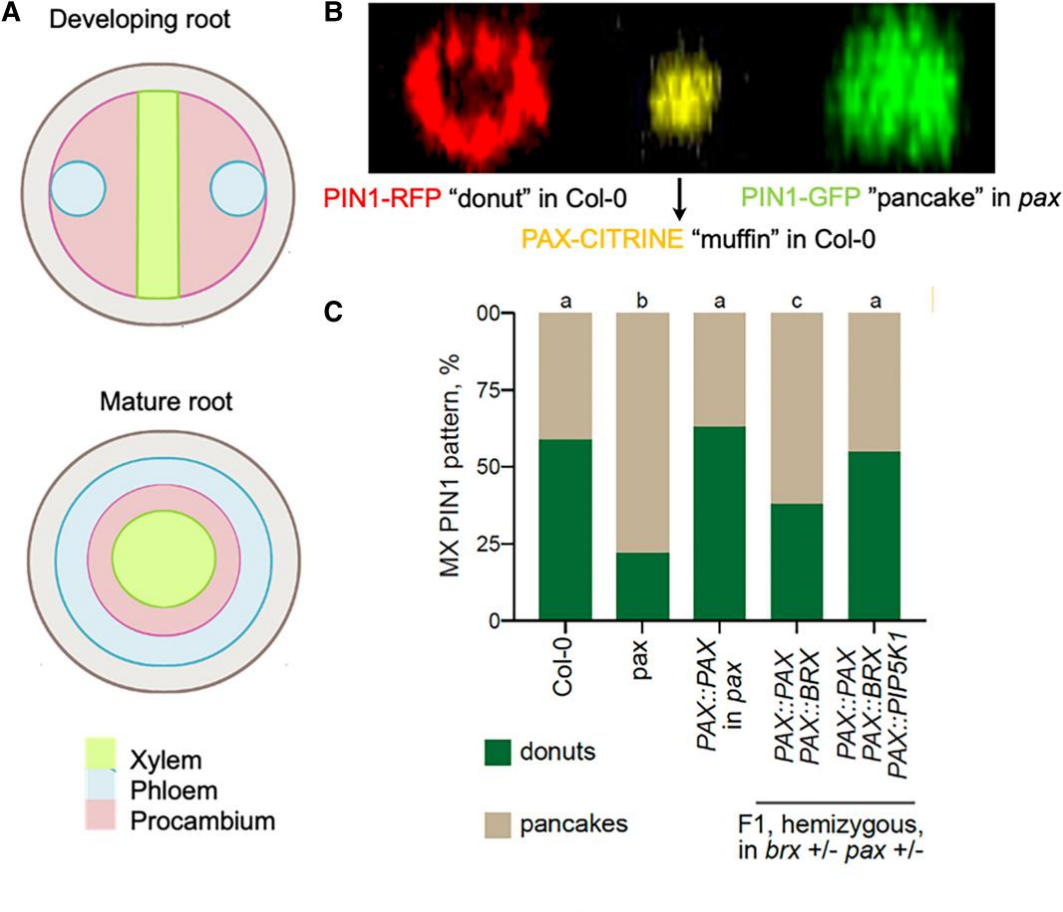
Protophloem cells and *PIN* and *PAX* expression patterns. **A)** Organization of the protophloem and protoxylem cells and in young and mature roots. **B)** PIN1 and PAX pattern at the PM of wild-type and mutant roots. **C)** Proportion of PIN1 donut and pancake pattern in the developing metaxylem (MX) of various inducible lines compared to Col-0 and *pax*. B and C adapted from Aliaga Fandino et al. (2024), Figures 1A and 2F.

The working model for how auxin flux is modulated during PPSE differentiation is as follows. When intracellular auxin levels are high, the kinase PROTEIN KINASE ASSOCIATED WITH BRX (PAX) phosphorylates PIN and activates PIN-mediated auxin efflux. When auxin levels drop due to auxin efflux from the cell, the PPSE-expressed BREVIS RADIX (BRX) associates with PAX and inhibits it from activating PIN-mediated auxin efflux. Reduced auxin efflux replenishes intracellular auxin and enables kinase-mediated activation of PAX and the PAX-mediated phosphorylation and displacement of BRX from the PM (Marhava et al. 2018). The uninhibited PAX phosphorylates PIN, and PIN-mediated auxin efflux is restored. BRX and PAX are further stabilized by PIP5 K (Wang et al. 2023), which reinforces PAX polarity and likely creates conditions for clathrin-mediated endocytosis of PINs through promoting PI(4,5)P2 formation.

The self-reinforcing BRX-PAX-PIP5K rheostat resembles a “muffin” within the PIN1 minima that forms at the center of the rootward membrane. Due to the PIN1 minima at the center, the PIN1 pattern resembles a donut (Fig. B). Not only is this donut shape of PIN1 at the rootward membrane unique to the PPSE, PIN1 minima is abolished and takes on a more pancake-like conformation in *pax*, *brx*, and *pip5k* mutants (Marhava et al. 2020). In their follow-up study, Aliaga Fandino et al. (2024) ectopically express the BRX-PAX-PIP5K module in the developing phloem and xylem to tease apart the precise function of each individual component during the establishment of PIN polarity and auxin flow during vascular differentiation. The authors detected the PIN1 phosphorylation site and donut shape when they induced PAX in a *pax* mutant background. Using an estradiol-inducible system, the authors note that PIN1 localization is highly dynamic and shifts from donut to pancake upon the induction of endocytosis blockers. As expected, PIN1 assumed a pancake-like shape when BRX was induced in *brx* and dampened by the addition of PIP5K. Direct evidence for PIN1 endocytosis within the donut hole had been lacking until now. Now the authors even visualize PIN1 internalization originating from the PAX “muffin” and establish the importance of PAX in phosphorylating PIN1 and enabling its turnover.

During the development of the vasculature, PPSE differentiation is followed by differentiation of protoxylem cells at the central axis that give rise to the xylem (Fig. A). Because auxin levels and members of PIP5K are important for xylem differentiation (von der Mark et al. 2022), Aliaga Fandino et al. (2024) pondered if the BRX-PAX-PIP5K module could also help the developing xylem to differentiate. Despite being primarily expressed in PPSE, the ectopic expression of BRX had the same inhibitory effect on PAX in the developing xylem. Increasing PAX expression altered xylem development and differentiation and was in turn suppressed through the ectopic expression of BRX. When the entire BRX-PAX-PIP5K module was ectopically expressed, auxin levels increased, the xylem differentiated, and BRX's inhibitory effect on PAX was dampened (Fig. C). These findings support the unanimous importance of cellular auxin homeostasis and efflux machinery in regulating differentiation at the vasculature.

## Dive Curated Terms

The following phenotypic, genotypic, and functional terms are of significance to the work described in this paper:

brx Gramene: AT1G31880
brx Araport: AT1G31880

PIN1 Gramene: AT1G73590
PIN1 Araport: AT1G73590

PROTOPHLOEM AmiGo: PO:0006077
